# High-throughput olfactory conditioning and memory retention test show variation in Nasonia parasitic wasps

**DOI:** 10.1111/j.1601-183X.2012.00823.x

**Published:** 2012-08-14

**Authors:** K M Hoedjes, J L M Steidle, J H Werren, L E M Vet, H M Smid

**Affiliations:** † Laboratory of Entomology, Plant Sciences Group, Wageningen UniversityWageningen, The Netherlands; ‡ Institut für Zoologie, Fachgebiet Tierökologie, Universität HohenheimStuttgart, Germany; § Department of Biology, University of RochesterRochester, NY, USA; ¶ Department of Terrestrial Ecology, Netherlands Institute of Ecology (NIOO-KNAW)Wageningen, The Netherlands

**Keywords:** Adaptation, conditioning, electroantennogram, high-throughput, learning, memory, Nasonia, olfactometer, parasitoid wasp, T-maze

## Abstract

Most of our knowledge on learning and memory formation results from extensive studies on a small number of animal species. Although features and cellular pathways of learning and memory are highly similar in this diverse group of species, there are also subtle differences. Closely related species of parasitic wasps display substantial variation in memory dynamics and can be instrumental to understanding both the adaptive benefit of and mechanisms underlying this variation. Parasitic wasps of the genus Nasonia offer excellent opportunities for multidisciplinary research on this topic. Genetic and genomic resources available for Nasonia are unrivaled among parasitic wasps, providing tools for genetic dissection of mechanisms that cause differences in learning. This study presents a robust, high-throughput method for olfactory conditioning of Nasonia using a host encounter as reward. A T-maze olfactometer facilitates high-throughput memory retention testing and employs standardized odors of equal detectability, as quantified by electroantennogram recordings. Using this setup, differences in memory retention between Nasonia species were shown. In both Nasonia vitripennis and Nasonia longicornis, memory was observed up to at least 5 days after a single conditioning trial, whereas Nasonia giraulti lost its memory after 2 days. This difference in learning may be an adaptation to species-specific differences in ecological factors, for example, host preference. The high-throughput methods for conditioning and memory retention testing are essential tools to study both ultimate and proximate factors that cause variation in learning and memory formation in Nasonia and other parasitic wasp species.

Learning and memory have been shown in a large number of animal species, with a focus on a few well-established model species, including the marine snail (a mollusc), the fruit fly and honey bee (insects), several bird species and mammals (most importantly mouse and rat) (Bottjer & Johnson 1997; Chen & Tonegawa 1997; Eisenhardt 2006; Margulies *et al*. 2005; Reissner *et al*. 2006). The importance of learning is reflected by strikingly similar features of memory formation in this diverse group, such as the effects of massed or spaced conditioning, as well as highly conserved neural and genetic pathways that underlie this trait (Dubnau 2003). Nonetheless, differences can be observed as learning is shaped by differences in ecology between animal species (Hoedjes *et al*. 2011). Furthermore, memory dynamics can vary within a species depending on the type of conditioning (Burke & Waddell 2011; Nakatani *et al*. 2009). Variation is determined by factors such as quantity and quality of the reward or punishment, and reliability of learned cues (Hoedjes *et al*. 2011). In-depth studies on a larger number of species and on different types of conditioning are needed to understand variation in learning and memory. Parasitic wasps can be instrumental for understanding this variation.

Several wasp species are ecologically and behaviorally well studied, are known to learn environmental cues readily (Vet *et al*. 1995) and display substantial interspecific and intraspecific variation in memory dynamics (van den Berg *et al*. 2011; Smid *et al*. 2007; Tamo *et al*. 2006). Learning plays an important role in optimizing foraging efficiency, and thus reproductive success, of female wasps searching for hosts (Papaj & Vet 1990). Parasitic wasps therefore feature an alternative type of appetitive conditioning, based on the reward that a female wasp experiences when finding and ovipositing in a host, so-called oviposition learning. This study is the first to show differences in oviposition learning between species of the genus *Nasonia*. Especially these species are excellent for comparative, multidisciplinary studies of variation in learning and memory. Many aspects of the biology of *Nasonia vitripennis*, *Nasonia giraulti* and *Nasonia longicornis* have been studied, and the species are known to differ in host range and host preference (Darling & Werren 1990; Desjardins *et al*. 2010). Furthermore, their genomes have been sequenced, and there are several unique genetic tools available (Werren & Loehlin 2009; Werren *et al*. 2010). This provides opportunities to test hypotheses on how ecological differences may result in different memory dynamics as well as studying the genetic basis of this variation.

Previous studies showed learning in *N. vitripennis* but involved laborious methods for conditioning and testing memory retention (Oliai & King 2000; Schurmann *et al*. 2009). In *Drosophila*, the availability of standardized, high-throughput methods for both conditioning and memory testing (Tully & Quinn 1985) was a prerequisite for the immense success of this model species. This article describes the development of both high-throughput olfactory conditioning and a memory retention test for the *Nasonia* model system. This setup was successfully used for *N. vitripennis*, *N. longicornis* and *N. giraulti*.

## Materials and methods

### Nasonia strains and maintenance

Cultures of *Nasonia* were reared in polystyrene rearing vials (dimensions 28.5 x 95 mm) with foam stoppers (Genesee Scientific, San Diego, CA, USA) in a climate cabinet under a constant temperature (25°C) and a photoperiod of 16:8 (L/D). The wasps were reared on *Calliphora vomitoria* pupae, which were obtained as maggots (Kreikamp, Hoevelaken, the Netherlands) and allowed to pupate at room temperature and subsequently stored in a fridge for a maximum of 1 week. Inbred strains of *N. vitripennis* (AsymC), *N. giraulti* [RV2x(U)] and *N. longicornis* [IV7(U)] were used for the experiments (Werren *et al*. 2010). Both females and males were collected on the day of emergence to ensure mating and were kept in vials with access to honey and water. Females were used in experiments between 1 and 3 days after emergence.

### Electroantennogram analysis

Odors that were expected to be neutral stimuli to *Nasonia* were chosen for the experiments. Vanilla and chocolate extract (Natural Chocolate extract and 2× Royal Brand Bourbon Vanilla extract; Nielsen-Massey Vanillas Intl., Leeuwarden, the Netherlands) are watery extracts that produce complex odor blends. Electroantennogram (EAG) analysis was performed to confirm that all three species had a similar sensitivity to these two odors. A large difference in sensitivity to one of the odors would make it more difficult for wasps to detect both odors in the olfactometer and could hinder the detection of memory retention. We used an EAG setup as described previously (Smid *et al*. 2002). Odor blends were diluted in water to 1%, 10% and 100%, and glass capillaries (Stuart SMP1/4, inner diameter: 1.3 mm, length adjusted to 30 mm; Bibby Scientific, Staffordshire, UK) were filled with these odors. The glass capillaries were subsequently placed in a Pasteur pipet and attached to the wall of the pipet with double-sided adhesive tape to ensure that both ends are exposed to the air in the Pasteur pipet. The resulting odor cartridge was then sealed with parafilm until use.

*Nasonia* wasps were decapitated and the very tip of the antenna was cut with a scalpel. The tip of the antenna was then brought into contact with the glass recording electrode of the EAG setup, whereas the head, with a part of the prothorax attached, was connected to the ground electrode, as described previously. Natural almond extract (Nielsen-Massey Vanillas Intl.) 10% diluted in water was used as a standard odor. All measurements were corrected for responses to a blank odor stimulus (pure water) and normalized to the standard odor as described previously (Smid *et al*. 2002).

### Olfactory conditioning assay

This olfactory conditioning assay is a simulation of natural behavior, in which female wasps likely use odor cues to find suitable host patches and then localize hosts in the patch (Whiting 2010). The long-range searching behavior is omitted in this assay and female wasps are placed in close contact with the hosts instead. The female wasps will immediately perceive the odor and will encounter a host quickly thereafter; for this reason, the conditioning assay is considered a form of classical (Pavlovian) conditioning. The aim of the conditioning procedure was to associate a rewarding host encounter (unconditioned stimulus, US) with one of the two odors, vanilla or chocolate (conditioned stimulus, CS+), followed by exposure to the other odor without reward (CS−). This differential conditioning procedure was carried out in a reciprocal manner, where each group of 48 wasps was divided into two parts, one receiving a CS+ with chocolate followed by a CS− with vanilla, and the other a CS+ with vanilla and a CS− with chocolate. CS+ conditioning was adapted from Schurmann *et al*. (2012) and was performed at room temperature in wells of a 12-well microtiter plate (Greiner Bio-One, Alphen aan den Rijn, the Netherlands), which allows individual observations during the conditioning procedure. Two *C. vomitoria* pupae (US) and a piece of filter paper (0.75 cm^2^) with 1 µl vanilla or chocolate extract (CS) were placed in a well, before one female wasp was released from an aspirator into the well, which was then closed (not airtight) with a plastic cap (protection plug, 21.7 mm diameter; Skiffy, Amsterdam, the Netherlands). A wasp can immediately smell the odor and, because of the small size of the well, will encounter a host quickly thereafter. The wasp was then left for 1 h, in which she typically drills into a host pupa, forms a feeding tube and starts to feed from it. Wasps that did not start drilling within the first 30 min of the training were noted and removed from the experiment after 1 h. All wasps that had shown drilling behavior were then gently transferred to an empty rearing vial and kept here, as a group, for 15 min. A glass capillary with one closed end (ID 1.3 mm, length adjusted to 30 mm; Fisher Emergo, Landsmeer, The Netherlands) was filled with the complementary odor (CS−) using a syringe and was then placed in the vial. Wasps were exposed to this odor without a reward for 15 min. Earlier research on *N. vitripennis* has shown that presenting the insect with an odor (CS−) after the reward (a host experience) results in decreased attraction to that odor (Schurmann *et al*. 2009). Differential conditioning with two odors (CS+ and CS−) was therefore expected to result in a stronger preference shift toward the CS+ than a conditioning with CS+ only, similar to the result in fruit flies (Tully & Quinn 1985). When conditioning was finished, wasps were transferred to rearing vials with access to honey and water and kept in a climate cabinet under a constant temperature (25°C) and a photoperiod of 16:8 (L/D) until testing. Groups of 48 (two reciprocal groups of 24 wasps) *N. vitripennis*, *N. longicornis* or *N. giraulti* were conditioned as described above. Each reciprocal group of 24 wasps was then divided in two groups of 12 wasps at 4 (±0.5), 24 (±1), 48 (±1), 72 (±1), 96 (±1) or 120 (±1) h after conditioning to test memory retention (see below). This was repeated five times on different days, resulting in 20 groups (10 per reciprocal conditioning) per data point. In addition, groups of *N. vitripennis* were ‘conditioned’ with the same procedure but without host reward to assess the effects of presentation of the odors alone. These wasps were tested 4 h (±0.5) after conditioning.

### Memory retention test

**Figure 1:**
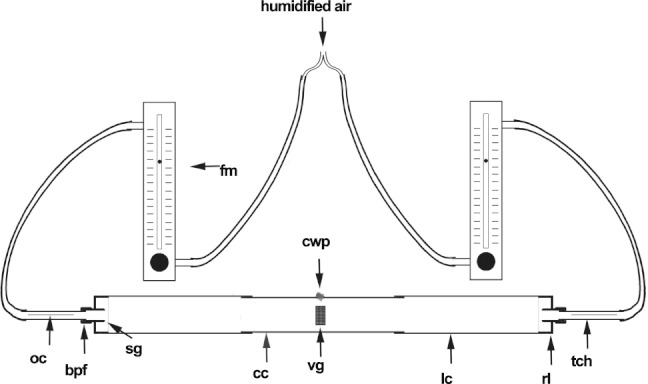
Schematic overview of the T-maze for Nasonia The T-maze consists of a central cylinder (cc) that slides into two lateral cylinders (lc). The cylinders are connected to a Teflon capillary holder (tch) with a brass press fit (bpf) fitted on a polyvinyl chloride (PVC), removable lid (rl). The Teflon capillary holders are holding the odor capillaries (oc) for odor supply. Humidified and charcoal-filtered air is blown into the T-maze via adjustable flow meters (fm) and can leave the T-maze via the ventilation grid (vg) in the central cylinder. Wasps are released through a hole in the center of the central cylinder and are allowed to move freely in the T-maze. Escape is prevented by closing the hole in the center with a cotton wool plug (cwp), netting on the ventilation grid and netting on the side grid (sg).

The T-maze olfactometer (Fig. 1) designed for testing memory retention in *Nasonia* was adapted and modified from the well-established T-maze designed for *Drosophila* (Tully & Quinn 1985). Wasps of the genus *Nasonia* are commonly observed to move by walking, making this setup suitable for this species. Differences in behavior between *Drosophilia* and *Nasonia* did require a number of modifications to the design. The T-maze for *Nasonia* was enlarged, because crowding of *Nasonia* wasps in smaller tubes resulted in fleeing from each other. The design for *Nasonia* does not include a training tube and sliding center as training was performed in microtiter plates as described above. The T-maze was made of Plexiglas and consisted of three tubes. Two lateral tubes (length: 20 cm and diameter: 4 cm) were connected to the center tube (length: 20 cm and diameter: 3.5 cm), which were attached by sliding them into each other. The distal ends of the tubes were connected to Teflon tubing for odor supply. Charcoal filtered, moisturized air (60–70% relative humidity) was blown into the apparatus with a flow rate of 100 ml/min on each side, which can leave the setup through ventilation slits in the middle tube. Polyamide netting (Monodur, PA 250; Nedfilter b.v., Almere, the Netherlands) prevented wasps from entering the Teflon tubing and ventilation slits. Capillaries filled with odors (chocolate or vanilla, respectively, as described above) were introduced in the Teflon tubing adjacent to the connection with the lateral Plexiglas tubes. Odor supply was adjusted for each species by the number of capillaries that were placed in the Teflon tube until naïve wasps distributed themselves approximately 50:50 when given a choice for chocolate and vanilla [groups of 12 (±2) wasps were released simultaneously, and 20 groups were tested on 5 different days]. For testing memory retention in *N. vitripennis* and *N. longicornis*, two capillaries of chocolate extract and two capillaries of vanilla extract were placed in the tubes. In the case of *N. giraulti*, four capillaries of chocolate extract and two capillaries of vanilla extract were placed in the tubes. The entire setup was shielded from environmental light and surrounded by white surfaces, and illumination was provided from above by LED strip illumination (Grandi ‘white’ 6000-6500K, 170 lm/m with 30 leds/m mounted against a white shelf 40 cm above the T-maze). During the run, a sheath of white paper (Satino, van Houtum, the Netherlands) shielded the T-maze from direct illumination.

Standard procedure involves testing memory for each of the two reciprocal pairs of up to 24 wasps in two runs to prevent crowding in the T-maze. Ten to twelve wasps were released into the middle part through a circular opening (8 mm diameter) using an aspirator, after which the opening was closed with a cotton wool plug. The memory retention test was performed at a temperature of 23.5 ± 1°C. Wasps that were released in the T-maze are allowed to move freely in the tubes for 10 min, after which their choice is recorded. Only the wasps that have entered one of the two arms were considered to have made a choice. Wasps that remain in the middle part of the T-maze are regarded as non-responding. On average, approximately 5–10% of the wasps do not respond in the test, and these wasps were ignored in the data analysis.

Experiments were performed to assess whether releasing wasps in groups of 12 (the number of wasps trained in one 12-well microtiter plate) affected the choice they made in the T-maze compared with individually released wasps in *N. vitripennis*. A total of 12 (±2) wasps trained on vanilla and chocolate (CS+) were tested individually 4 h (±0.5) after conditioning. Two reciprocal groups were reconstructed from the results of individually released wasps. This was repeated five times on different days. Memory retention was compared with wasps that had been tested in groups of 12 (±2) wasps 4 h (±0.5) after conditioning.

### Data analysis

Relative EAG responses were calculated as a percentage response compared with 10% almond. We used univariate analysis of variance (anova) to test whether there were differences in odor perception between the two odors, chocolate and vanilla, for each species and whether there were differences in relative sensitivity between the species. Naïve preferences toward the odors in the T-maze were analyzed by calculating percentage of wasps choosing vanilla for each group (*n* = 20). These percentages were tested with a *t*-test with a test value of 50.

Conditioning is expected to result in a shift in preference of the two groups of wasps toward the CS+. The difference in preference between two reciprocal groups was used as a measure for memory retention and is represented by the performance index (PI), comparable to the PI described by Tully *et al*. (1994). The first group has vanilla as CS+ and chocolate as CS−, and the second group has chocolate as CS+ and vanilla as CS−. The PI is calculated by subtracting the percentage of the second group choosing vanilla (CS−) from the percentage of the first group choosing vanilla (CS+): group 1 (CS+) − group 2 (CS−). If all trained wasps choose CS+, the difference between two reciprocal groups is at its maximum and the PI would be 100. This would represent perfect memory retention. When there is no memory retention, the two groups will choose similarly; this would result in a PI of 0. In order to monitor odor bias or preference after conditioning, an analysis was performed to observe if reciprocal groups showed a similar shift in preference toward the CS+ after conditioning. This was performed by subtracting the percentage of the second group choosing chocolate (CS+) from the percentage of the first group choosing vanilla (CS+). An equal shift in preference in both groups will result in a value of 0, which means that there is no bias toward one of the odors. All values from different time points after conditioning were taken together for each of the three species (*n* = 60), and a *t*-test was performed to test for odor bias after conditioning.

Two reciprocal groups of 24 wasps that were trained on 1 day were tested in two series of 12 wasps. Two reciprocal series of trained wasps were tested immediately after each other and a PI was calculated for these wasps. The setup was then turned 180° to average out the effect of external factors, and the two remaining series of wasps were tested. This results in two PIs per day. The experiment was repeated five times in total, resulting in 10 PIs. Normality and equal variances of the data were tested, and a *t*-test was used to test memory retention for each species at each time point. Univariate anova was used to test whether there were differences between species, between time points and whether these factors interact. PIs of wasps released in groups or individually (*N. vitripennis*, 4 h after training) were compared with an independent samples *t*-test. All statistical analyses were performed in SPSS, version 19 (IBM, Armonk, NY, USA).

## Results

### High-throughput olfactory conditioning assay for Nasonia

This study describes a conditioning assay in which female wasps of the genus *Nasonia* associate an odor (CS+) with a rewarding host encounter, in this case two *C. vomitoria* pupae. When a female wasp encounters a host pupa, she will touch the host with her antennae and she will subsequently start drilling into the puparium. In general, 85–100% of all females will initiate drilling within 30 min. When a female finishes drilling, she can build a feeding tube and feed from the host. No obvious differences were observed in drilling or feeding behavior between *N. vitripennis*, *N. longicornis* and *N. giraulti* in pilot experiments (results not shown). After associating an odor (CS+) with the rewarding host encounter, the wasps were exposed to the second odor without a reward (CS−). This conditioning step was found to improve PIs compared with training with the CS+ alone in pilot experiments (results not shown) and was therefore included in the procedure. Presentation of the odors alone, without a reward, does not result in significant memory formation (PI = −0.2 ± 5.5; *n* = 10, *t*_9_ = −0.038, *P* = 0.979). This conditioning assay can be used for all three species making it suitable for comparative studies on *Nasonia* spp. Training wasps in microtiter plates allows simultaneous conditioning of large numbers of individual wasps, while efficient individual monitoring remains possible.

### Choice and concentration of odors

**Figure 2:**
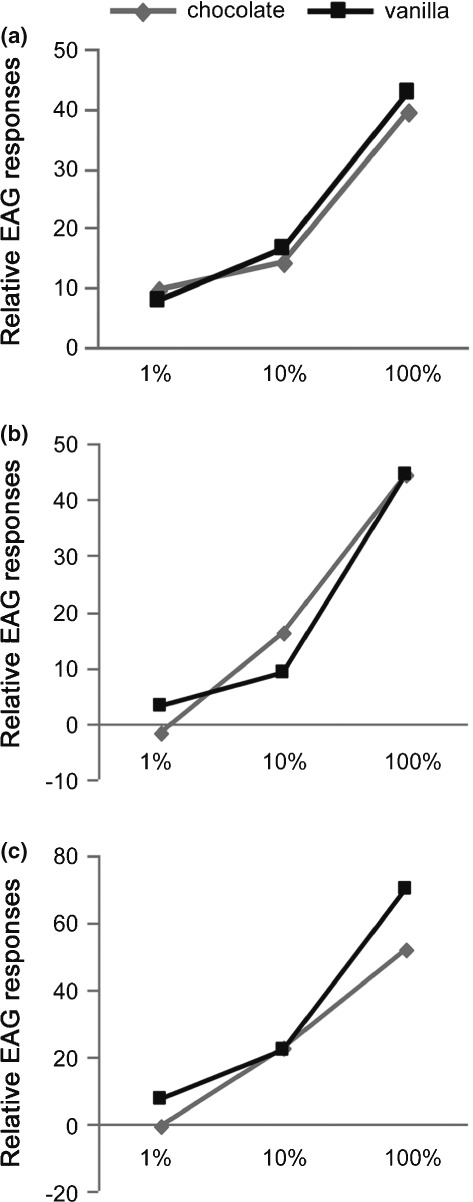
Electroantennogram responses Relative EAG responses of (a) N. vitripennis, (b) N. longicornis and (c) N. giraulti to different concentrations of chocolate and vanilla odor. All responses were corrected for blank with EAG response to 10% almond odor in water (*n* = 17 for N. vitripennis and *n* = 13 for N. longicornis and N. giraulti). There was a significant effect of concentration and species, but no significant effect of odor. None of the interactions was significant.

All three species responded in a dosage-dependent manner to both vanilla and chocolate odor. The EAG analyses (Fig. 2) showed that there was a significant effect of concentration as well as of species, showing that there are differences in relative responses between species as well as between different odor concentrations. There was, however, no significant effect of odor, which indicates that the responses to both odors are equal (odor: *F*_1,240_ = 0.979, *P* = 0.323; concentration: *F*_2,240_ = 89.343, *P* < 0.001; species: *F*_2,240_ = 4.177, *P* = 0.016; odor × concentration: *F*_2,240_ = 0.733, *P* = 0.481; odor × species: *F*_2,240_ = 0.752, *P* = 0.472; concentration × species: *F*_4,240_ = 1.62, *P* = 0.170; odor × concentration × species: *F*_4,240_ = 0.449, *P* = 0.773). Behavioral responses of unconditioned wasps toward vanilla and chocolate odor showed that when two capillaries of vanilla and chocolate were placed in the T-maze, both *N. vitripennis* (*t*_19_ = −0.292, *P* = 0.774) and *N. longicornis* (*t*_19_ = 0.158, *P* = 0.876) preferred the odors equally, resulting in a near 50:50 distribution. For *N. giraulti* (*t*_19_ = 0.737, *P* = 0.470), two capillaries of vanilla and four capillaries of chocolate resulted in a near 50:50 distribution. Both the results from EAG analyses and behavioral tests show that vanilla and chocolate are suitable odor sources for this assay.

### High-throughput memory retention test using the T-maze

**Figure 3:**
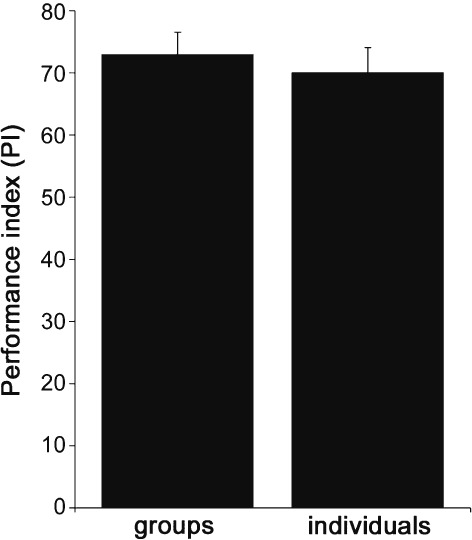
Effect of testing Nasonia vitripennis in the T-maze individually vs. a group of wasps Memory retention of individual and groups of 10–13 N. vitripennis wasps was compared 4 h after conditioning to assess if testing groups of wasps simultaneously had an effect on the PIs. The PI of wasps tested in groups is 73.0 ± 3.5 (*n* = 10); the PI of individually tested wasps is 70.1 ± 4.0 (*n* = 5). There is no significant difference between these scores.

A T-maze olfactometer was designed to allow high-throughput testing of memory retention in *Nasonia*. Groups of wasps can be tested simultaneously in this olfactometer, greatly reducing time that is needed to test a certain number of wasps. Experiments were performed to determine whether testing wasps in groups had any effect on the choice behavior in the T-maze. Although no apparent interference of wasps was observed, i.e. wasps did not appear to avoid or follow each other, it may be possible that a wasp is influenced by choices that other wasps of a group make. Comparisons between PIs calculated from reciprocal groups of 12 (±2) wasps tested either individually or as groups showed that there was no effect on PI (group tested: PI = 73.0, *n* = 10; individually tested: PI = 70.1, *n* = 5; *t*_13_ = 0.517, *P* = 0.614) (Fig. 3).

In the T-maze, the wasps walk toward one of the two odor sources. *Nasonia vitripennis*, *N. longicornis* and *N. giraulti* were observed to walk readily into the two lateral tubes, and only a minority of the wasps did not leave the middle tube. This T-maze setup can be considered suitable for all *Nasonia* species.

### Memory retention in Nasonia

**Figure 4:**
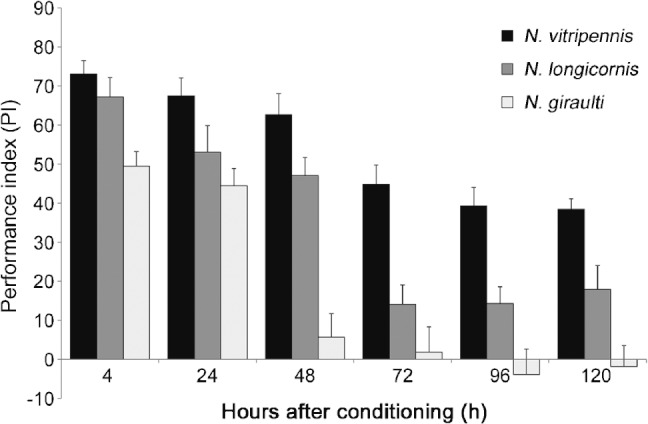
Memory retention of Nasonia after a single conditioning Memory retention of N. vitripennis, N. longicornis and N. giraulti was tested in the T-maze at 4, 24, 48, 72, 96 and 120 h (*n* = 10) after the oviposition conditioning procedure. All three species had significant memory at 4 and 24 h. Both N. vitripennis and N. longicornis have significant memory at 48, 72, 96 and 120 h after training as well, whereas N. giraulti did not. The PIs of the three species differ from each other and differ between time points. Species and time points also interact.

Memory retention was tested for *N. vitripennis*, *N. longicornis* and *N. giraulti* at 4, 24, 48, 72, 96 and 120 h after one olfactory conditioning (Fig. 4). All three species were found to have a significant retention of memory at 4 and 24 h after conditioning. These results show that both the conditioning assay and the memory retention test can be used successfully for these *Nasonia* species. After 48 h, *N. giraulti* had lost its memory, whereas both *N. vitripennis* and *N. longicornis* have memory up to at least 120 h after conditioning (Fig. 4 and Table 1). The memory dynamics of the three species differ from each other (*F*_2,174_ = 12.649, *P* < 0.001) and the PI decreases over time (*F*_1,174_ = 177.556, *P* < 0.001). There was no significant interaction of species and time (*F*_2,174_ = 2.349, *P* = 0.098). These results from the analyses show that both *N. vitripennis* and *N. longicornis* have a long-lasting memory retention after a single conditioning, although their PIs decrease over time. *Nasonia giraulti* only has a relatively short memory retention up to 24 h after a similar conditioning.

**Table 1:** Memory retention of Nasonia after a single conditioning (statistics)

	*N. vitripennis*			*N. longicornis*			*N. giraulti*		
Hours (h)	PI	*t*_9_ value	*P* value	PI	*t*_9_ value	*P* value	PI	*t*_9_ value	*P* value
4	73.0	20.92	<0.001^*^^*^^*^	67.2	13.47	<0.001^*^^*^^*^	49.4	13.21	<0.001^*^^*^^*^
24	67.5	14.62	<0.001^*^^*^^*^	53.0	7.77	<0.001^*^^*^^*^	44.5	10.30	<0.001^*^^*^^*^
48	62.7	11.70	<0.001^*^^*^^*^	47.0	10.21	<0.001^*^^*^^*^	5.7	0.94	0.372 n.s.
72	44.7	8.93	<0.001^*^^*^^*^	14.1	2.88	0.018^*^	1.8	0.28	0.789 n.s.
96	39.3	8.28	<0.001^*^^*^^*^	14.3	3.36	0.008^*^^*^	−3.9	−0.60	0.564 n.s.
120	38.4	14.42	<0.001^*^^*^^*^	17.9	2.94	0.016^*^	−1.9	−0.35	0.734 n.s.

A *t*-test was used to test memory retention of each species at 4, 24, 48, 72, 96 and 120 h (*n* = 10) after the oviposition conditioning procedure.

n.s., not significant.

Asterisks indicate the level of significance (^*^^*^^*^P < 0.001, ^*^^*^ < *P* < 0.01, ^*^0.01 < *P* < 0.05).

The odor preference was 50:50 for vanilla and chocolate in naïve animals, but after conditioning a bias toward one of the two odors was found in all three *Nasonia* species (*n* = 60). Both *N. vitripennis* (8.9%, *t*_59_ = −3.508, *P* = 0.001) and *N. longicornis* (6.0%, *t*_59_ = −2.181, *P* = 0.033) have a slight bias toward chocolate odor after conditioning. *Nasonia giraulti* (7.1%, *t*_59_ = 2.493, *P* = 0.015) has a slight bias toward vanilla odor after conditioning. This result shows that the odor preference of the three species changes after conditioning, emphasizing the importance of using a reciprocal setup for memory retention tests.

## Discussion

This study presents a novel method for high-throughput olfactory conditioning and memory retention testing of *Nasonia*. The olfactory conditioning assay was used to investigate the association of vanilla or chocolate odor (CS+) with the reward of finding a host (US) in female wasps of *N. vitripennis*, *N. longicornis* and *N. giraulti*. All three *Nasonia* species could be conditioned using a similar protocol, allowing a good comparison of learning and memory between the species. The US consisted of multiple components in this assay; the female wasp first touches the host with her antenna and thereby will perceive chemical information inducing drilling behavior. She drills a hole in the puparium and will use her ovipositor to find the host and assess its quality; then she feeds from the pupa, which is required for egg production (Whiting 2010). Previous studies have shown that drilling alone is sufficient for *N. vitripennis* to form an anesthesia-resistant memory that lasts up to 4 days (Schurmann *et al*. 2009). Both drilling and host feeding result in a long-term, protein synthesis-dependent memory (LTM) that lasts for at least 6 days (Schurmann *et al*. 2012). This shows that these two components of the US affect the strength of the memory differently. Access to the host for 1 h typically enables wasps to obtain multiple experiences consisting of drilling and host feeding, but actual oviposition does not occur yet (Schurmann *et al*. 2012). It will be interesting to study how drilling alone, drilling and host feeding and oviposition affect memory retention in *N. longicornis* and *N. giraulti* as well.

The T-maze olfactometer facilitates comparative memory retention tests of the three *Nasonia* species using the same odors. The odor concentrations were chosen to result in a 50:50 distribution of naïve animals. For *N. giraulti*, a higher concentration of chocolate odor was required than for *N. vitripennis* and *N. longicornis* to achieve this equal distribution. This difference between *N. giraulti* and the other two species may be the result of a slight, but insignificantly higher antennal sensitivity for vanilla compared with chocolate (Fig. 2). After conditioning, the preference of *N. vitripennis* and *N. longicornis* shifts, slightly, toward chocolate odor, whereas the preference of *N. giraulti* shifts toward vanilla odor. A similar preference shift was also found in the parasitic wasp *Leptopilina heterotoma* and may be a result of a change in sensitivity in olfactory receptor neurons due to conditioning (Vet *et al*. 1990). EAG analyses before and after conditioning can elucidate this question.

Both the conditioning procedure and testing memory retention in the T-maze olfactometer are high-throughput methods compared with individual conditioning and testing of wasps (van den Berg *et al*. 2011; Huigens *et al*. 2009; Schurmann *et al*. 2009). This makes it possible to determine memory retention of a group of wasps more accurately. The average PIs in this study were calculated from a sample size of 10, and the standard errors were between 2.5 and 7. The PI is therefore a highly reproducible measure for memory allowing detection of small differences in memory retention. We expect that the T-maze can be used for many other parasitic wasps as well, especially those that are known to exhibit olfactory microhabitat and host location by walking, such as parasitic wasps of *Drosophila* (Kaiser *et al*. 2009; Vet 1985) and *Lariophagus* (Muller *et al*. 2006). The T-maze may also be adapted to conduct high-throughput studies on olfactory responses of *Nasonia* or other parasitic wasps in the context of host location, e.g. comparable to Turlings *et al*. (2004). Being able to perform high-throughput studies on learning and olfaction on more species of parasitic wasps will greatly accelerate studies of variation in learning and memory.

This study shows differences in memory retention between *N. vitripennis* (AsymC), *N. longicornis* [IV7(U)] and *N. giraulti* [RV2x(U)]. This result may indicate that there are differences in memory retention between populations or species in this genus. More strains of these species need to be tested to investigate this variation on a wider scale. In both *N. vitripennis* and *N. longicornis*, memory was observed up to at least 5 days, although the PIs decreased over time. This may be long-term memory, which was also observed in another strain of *N. vitripennis* when conditioned with a comparable procedure (Schurmann *et al*. 2012). In contrast, no memory was present after 1 day in *N. giraulti*, showing that the memory dynamics of *N. giraulti* differ from the other two species. The significance of the differences between the three *Nasonia* strains may become clear when analyzing memory dynamics using specific memory inhibitors, as previously shown for *Cotesia* parasitic wasps (Smid *et al*. 2007).

Future studies can focus on ultimate and proximate factors that cause this variation in parasitic wasps. Ecological differences such as host range and host distribution are considered important ultimate factors that determine learning rate or memory dynamics (Hoedjes *et al*. 2011). Species or populations that have a wide host range and a wide host distribution may need to divide their attention over a wide variety of cues. Learning may be important to limit their ‘search image’ and focus only on a specific type of habitat or host that is available (Dukas 1998; Ishii & Shimada 2010). *Nasonia vitripennis*, and to a lesser degree *N. longicornis*, are considered to be generalist species that will parasitize a wide range of fly species, most importantly from the families Sarcophagidae and Calliphoridae, in a number of distinct habitats, such as manure, decaying carcasses and birds' nests. A wider host range may explain why the strains of *N. vitripennis* and *N. longicornis* tested in this study form a long-lasting memory after only a single learning experience. *Nasonia giraulti* is considered a specialist of Protocalliphora spp. in birds' nest and may rely more on innate preferences, e.g. for bird nest-specific odors (Darling & Werren 1990; Ings *et al*. 2009; Stephens 1993). Memory dynamics can also vary depending on the encountered host species. Certain host species are reliably associated with certain cues or habitats, whereas other species do not have such a reliable association. Differences in host preference or host suitability may also result in differences in host reward value for a female wasp (Hoedjes *et al*. 2011). Several studies have addressed aspects of *Nasonia* ecology, including natural host range, host and wasp distribution, host preference and host suitability (Darling & Werren 1990; Desjardins *et al*. 2010; Peters & Abraham 2004; Pimentel 1966; Rivers & Denlinger 1995). However, few of these studies have made a comparison between different *Nasonia* species or populations, and none has evaluated the effect of ecological factors on learning rate or memory dynamics. Such studies are necessary to understand which ecological factors shape learning and memory in *Nasonia*.

Wasps of the genus *Nasonia* are also excellent organisms to provide understanding of proximate factors that underlie differences in learning and memory. First of all, the genomes of three *Nasonia* species, *N. vitripennis*, *N. giraulti* and *N. longicornis*, are fully sequenced and partially annotated (Werren *et al*. 2010). Genetic tools for *Nasonia* include a number of arrays, such as a tiling microarray and comparative genomic hybridization mapping arrays, as well as detailed genetic and molecular marker maps. Another important characteristic of the *Nasonia* system is the possibility to interbreed different species. This allows backcrossing of loci that are involved in differences in learning and memory into another species of *Nasonia* (Werren & Loehlin 2009). A combination of these tools will allow one to pinpoint species-specific differences in genetic pathways causing differences in learning and memory. Next to genetic differences, neural pathways may also differ. Comparative studies in *Nasonia* can focus on the organization of groups of neurons or entire brain regions similar to studies in parasitic wasps of the genus *Cotesia*. Immunolabeling may, for example, show differences in the number or arborization patterns of reward neurons (Bleeker *et al*. 2006). Construction of 3D models of the brain and individual brain regions will provide insight into overall organization of the brains of different species (Smid *et al*. 2003). A combination of genetic and neural studies can provide extensive understanding of the mechanisms that cause differences in learning and memory in the genus *Nasonia*. It is expected that there is a large homology in genetic and neural pathways between these species and well-studied model insect species, such as fruit flies and bees (Dubnau 2003). The large amount of knowledge gained for research on these model insect will likely benefit studies in *Nasonia*.

We argue in this study that wasps of the genus *Nasonia* offer excellent opportunities for integrative studies on ultimate and proximate factors that cause variation in learning and memory formation. The novel olfactory conditioning assay and T-maze olfactometer for testing memory retention, presented in this study, facilitate high-throughput studies in *Nasonia* wasps. This setup may be used for studies on learning or olfaction in other parasitic wasps that locate their hosts by walking as well.
